# Rescue of germ cells in *dnd* crispant embryos opens the possibility to produce inherited sterility in Atlantic salmon

**DOI:** 10.1038/s41598-020-74876-2

**Published:** 2020-10-22

**Authors:** Hilal Güralp, Kai O. Skaftnesmo, Erik Kjærner-Semb, Anne Hege Straume, Lene Kleppe, Rüdiger W. Schulz, Rolf B. Edvardsen, Anna Wargelius

**Affiliations:** 1 Institute of Marine Research, Bergen, Norway; 2 Faculty of Fisheries and Protection of Waters, South Bohemian Research Center of Aquaculture and Biodiversity of Hydrocenoses, University of South Bohemia in Ceske Budejovice, Zátiší 728/II, 389 25 Vodňany, Czech Republic; 3 Department of Biology, Faculty of Science, Utrecht University, Padualaan 8, 3584 CH Utrecht, The Netherlands

**Keywords:** Biotechnology, Cell biology, Genetics, Molecular biology

## Abstract

Genetic introgression of escaped farmed Atlantic salmon (*Salmo salar*) into wild populations is a major environmental concern for the salmon aquaculture industry. Using sterile fish in commercial aquaculture operations is, therefore, a sustainable strategy for bio-containment. So far, the only commercially used methodology for producing sterile fish is triploidization. However, triploid fish are less robust. A novel approach in which to achieve sterility is to produce germ cell-free salmon, which can be accomplished by knocking out the *dead-end* (*dnd*) gene using CRISPR-Cas9. The lack of germ cells in the resulting *dnd* crispants, thus, prevents reproduction and inhibits subsequent large-scale production of sterile fish. Here, we report a rescue approach for producing germ cells in Atlantic salmon *dnd* crispants. To achieve this, we co-injected the wild-type (wt) variant of salmon *dnd* mRNA together with CRISPR-Cas9 constructs targeting *dnd* into 1-cell stage embryos. We found that rescued one-year-old fish contained germ cells, type A spermatogonia in males and previtellogenic primary oocytes in females. The method presented here opens a possibility for large-scale production of germ-cell free Atlantic salmon offspring through the genetically sterile broodstock which can pass the sterility trait on the next generation.

## Introduction

As the global expansion of the salmon aquaculture industry rapidly continues, the aim of optimizing aquaculture methods to improve food production while reducing the environmental impacts of the industry is of growing demand. The salmon industry is currently threatened by parasites such as sea lice and escapees, which negatively affect wild populations by interbreeding^1,2^. Using sterile salmon in production can prevent such unwanted genetic introgression. Since sterile salmon missing the *dnd* gene also do not produce sex steroids^3^, the drawbacks associated with increasing sex steroid levels, such as reduced growth, lower flesh quality and higher susceptibility to disease^4^, can be avoided as well. In addition, sterility in production fish safeguards Intellectual Property Rights (IPR) for breeding companies.

Currently, the only method available to sterilize commercial-scale numbers of salmon is triploidization^5,6^. However, triploid salmon are generally more sensitive to suboptimal rearing environments making them prone to skeletal deformities^7,8^ and less tolerant to rising seawater temperature^9^. Furthermore, despite the fact that triploid male salmon are sterile, they can – in contrast to *dnd* knockout fish – still produce sex steroids and hence compete with wild salmon males on breeding grounds, thereby threatening the fecundity of wild females^10^. New methods to induce sterility have been explored in model fish species, including the use of transgenic on–off systems and morpholino-mediated gene knock-down by bath treatment of embryo batches with the aim to apply to farmed fish^11^. However, suitable protocols implementing such approaches for Atlantic salmon are currently not available.

The Dead end (Dnd) protein plays a crucial role in germ cell formation in vertebrates, including fish^12^. Accordingly, morpholino-based *dnd* mRNA knockdown during early embryogenesis induced germ cell loss in several fish species, including medaka, loach, goldfish, sturgeon, zebrafish and salmon^13–18^. Also in Atlantic salmon, loss of *dnd* gene leads to germ cell loss^19^. In contrast to previous studies that used morpholinos, in Atlantic salmon, CRISPR-Cas9 mediated targeted mutagenesis was applied to remove *dnd* gene function, to obtain a gene-edited germ cell-free (GCF) and hence sterile fish. Importantly, follow-up studies showed that GCF Atlantic salmon remained immature and did not undergo puberty^3^, which is not the case for triploid salmon males^20^. Yet, *dnd* deficient salmon lack germ cells and consequently cannot be used for breeding. One possibility to produce *dnd*^−/−^ fish from fertile parents, is to cross heterozygote (*dnd*^+/−^) mutants. However, this approach would only produce 25% *dnd*^−/−^ fish, and the homozygous mutants would have to be identified by cumbersome genotyping and subsequent sorting of the fish^14^. It is known that primordial germ cell (PGC) formation can be rescued by co-injection of a *dnd* morpholino and *dnd* mRNA in zebrafish^21^. However, in that study, *dnd* was transiently removed during early embryogenesis. In the present study, we developed a different approach involving the permanent removal of *dnd* function in order to produce large numbers of *dnd*^−*/*−^ sterile offspring. Since it is uncertain based on data available from mammals if germ cells will survive and form a normal gonad in a *dnd*^−/−^background^22–25^, this uncertainty was one of the focus points of the present study. On the other hand, if we can rescue germ cells in a *dnd*^−/−^ salmon and these fish form a functional gonad, this opens the possibility for the large-scale production of genetically sterile GCF fish.

In order to achieve germ cell rescue in Atlantic salmon, we co-injected the wild-type (wt) variant of salmon *dnd* (mRNA) together with CRISPR-Cas9 constructs targeting *dnd* into 1-cell stage embryos. We observed that one-year old rescued *dnd* crispants developed testes and ovaries containing germ cells, type A spermatogonia and previtellogenic primary oocytes, respectively.

## Results

### Identification of rescued gonads in juvenile salmon

In this study, two experiments were carried out with the aim to rescue germ cells in Atlantic salmon *dnd* crispants. In the first experiment, we mutated *dnd* while also adding *dnd* mRNA, combined with mutating *slc45a2*, allowing to externally identify mutated fish by their albino phenotype. In one-year old rescued fish, we sampled gonads from thirteen albino fish (6 ♀ and 7 ♂) (body weight (BW): 36.84 ± 17.26 g; length (L): 14.25 ± 2.44 cm) and two wt controls (1 ♀ and 1 ♂) (BW: 41.55 ± 2.95 g; L: 15.75 ± 0.75 cm) . Two of the six albino females displayed normal gross morphology of the gonad, with a clearly visible ovarian bulb indicating rescue of germ cell development (Supplementary Fig. S1 sample ID 19 and 22). The remaining four females displayed no ovarian bulb (Supplementary Fig. S1 sample ID 12, 18, 21, and 23). Histological analysis of the ovaries confirmed the presence and absence of germ cells, respectively, associated with the presence or absence of an ovarian bulb. Rescued females with an ovarian bulb showed an ovarian histology (Supplementary Fig. S1 sample ID 19 and 22), similar to immature wt females (Supplementary Fig. S1 sample ID 2), i.e. the ovary contained several previtellogenic follicles in the primary growth phase. The remaining four albino females, on the other hand, lacked germ cells in their ovaries (Supplementary Fig. S1 sample ID 12, 18, 21, and 23). Germ cells were not found in any of the seven sampled males, suggesting that *dnd* mRNA injection did not rescue germ cell development in these males (Supplementary Fig. S2). Sampling again four months later, gonad samples from four albino males (BW: 58.25 ± 21 g; L: 16.12 ± 2.08 cm), one albino female (BW: 30 g; L: 14 cm) (1 ♀ and 4 ♂) , and from two wt controls (1 ♀ and 1 ♂) (male BW: 58 g; L:17.5 cm; female BW 150 g; L: 23 cm) were collected. The body weight and length of individuals together with other sampling details are presented in Supplementary Table S1. Histological analysis revealed that testis tissue from two of the four albino males contained spermatogonia (Supplementary Fig. S3 sample ID 58 and 83) while the remaining two males were GCF (Supplementary Fig. S3 sample ID 28 and 39). The morphology and histology of all sampled fish are shown in the Supplementary Figs. S1–3. In summary, from the potentially rescued fish (n = 79) (BW:47.68 ± 17.7 g; L: 15.84 ± 2.5 cm; n = 67), we sampled eighteen albino fish and four controls. We identified thirteen *dnd* crispants without germ cells and four *dnd* crispants with germ cells.

In the second experiment, we aimed to create a potential broodstock displaying normal skin pigmentation. Therefore, we omitted mutating *slc45a2*, when creating *dnd* crispants with potentially rescued germ cells due to co-injection of *dnd* mRNA. At 9 months post-fertilization (mpf), fin clips of 60 potentially rescued fish (BW: 34.95 ± 14.17 g; L: 13.58 ± 1.94 cm) and five controls (BW: 58 ± 16.64 g; L: 16.06 cm) (3 ♂ and 2 ♀) were sampled, 15% (5 ♂ and 4 ♀) of which had *dnd* mutations, as detected by High Resolution Melt (HRM) analysis.

**Figure 1:**
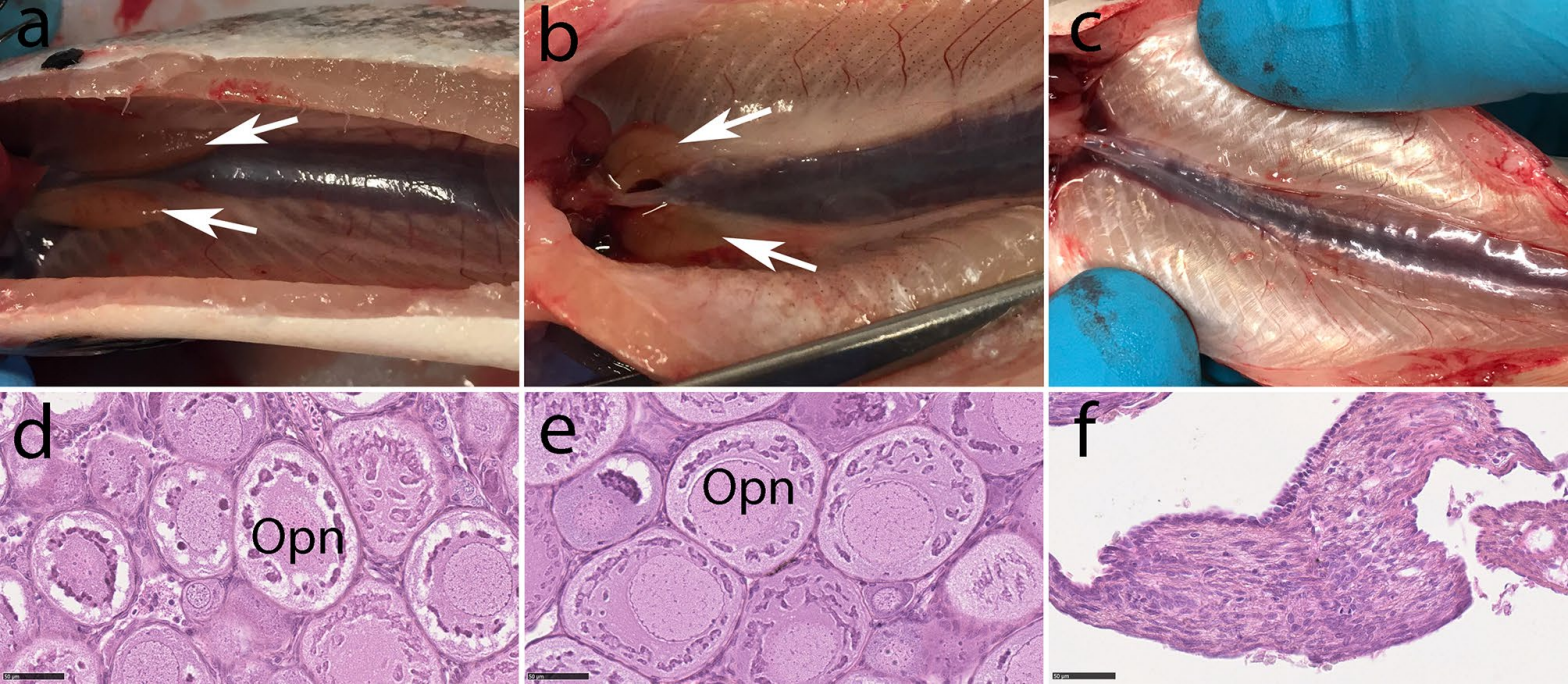
Macroscopic and microscopic anatomy of ovaries in one-year-old female wild type, rescued and germ cell-free *dnd* crispant Atlantic salmon. The gross morphology and histology of a control female is shown in the first row (**a**, **d**); the middle row shows a *dnd* crispant female with germ cells rescued by *dnd* mRNA injection (**b**, **e**); the last row shows a *dnd* crispant germ cell-free (GCF) female (**c**, **f**). The control fish shows an ovarian bulb (white arrow) (**a**) as does the rescued *dnd* crispant female (**b**), but the GCF *dnd* crispant albino female lacks an ovarian bulb (**c**). Primary oocytes in the perinucleolar stage (Opn) are observed in ovaries of the control wt female (**d**) and rescued *dnd* crispant female with oogonia (**e**), while no germ cells are observed in ovaries of *dnd* crispant albino female (**f**). Wt control female:160, rescued female: 139, GCF female: 23.

**Figure 2:**
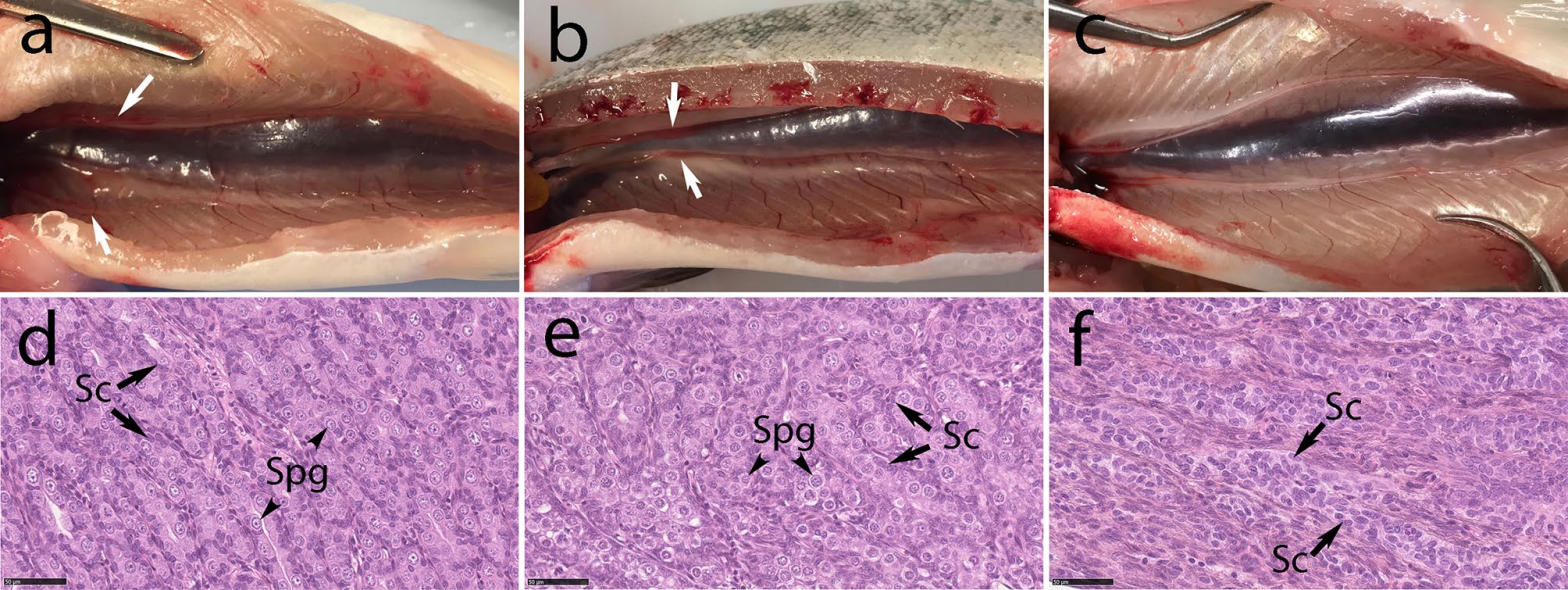
Macroscopic and microscopic anatomy of testes in 16-month-old male wild type, rescued and germ cell-free *dnd* crispant Atlantic salmon. The gross morphology and histology of a control male is shown on the first row (**a**, **d**); the middle row shows a *dnd* crispant male with germ cells rescued by *dnd* mRNA injection (**b**, **e**); the last row shows a *dnd* crispant GCF albino male (**c**, **f**). The testis of each males is shown by white arrows (a, b, c); The spermatogonia (Spg; black arrowhead) surrounded by Sertoli cells (Sc; black arrow) were observed in testis of the control wt male (**d**) and the rescued *dnd* crispant male (**e**) but not in testis of *dnd* crispant albino male (**f**). Wt control male: 162, rescued male: 148, GCF male: 16.

At 11 months post fertilization, histological analysis demonstrated that two of the four female *dnd* crispants had ovaries with germ cells (Fig. 1, Supplementary Fig. S4 sample ID 108 and 139). We also found that four of the five male *dnd* mutants were classified as immature based on gross morphology, and further confirmed to have spermatogonia by histology (Fig. 2, Supplementary Fig. S5 sample ID 130, 146, 148 and 150). The body weight and length of individuals at 11 mpf together with other sampling details are presented in Supplementary Table S2.

### Relative gene expression

**Figure 3:**
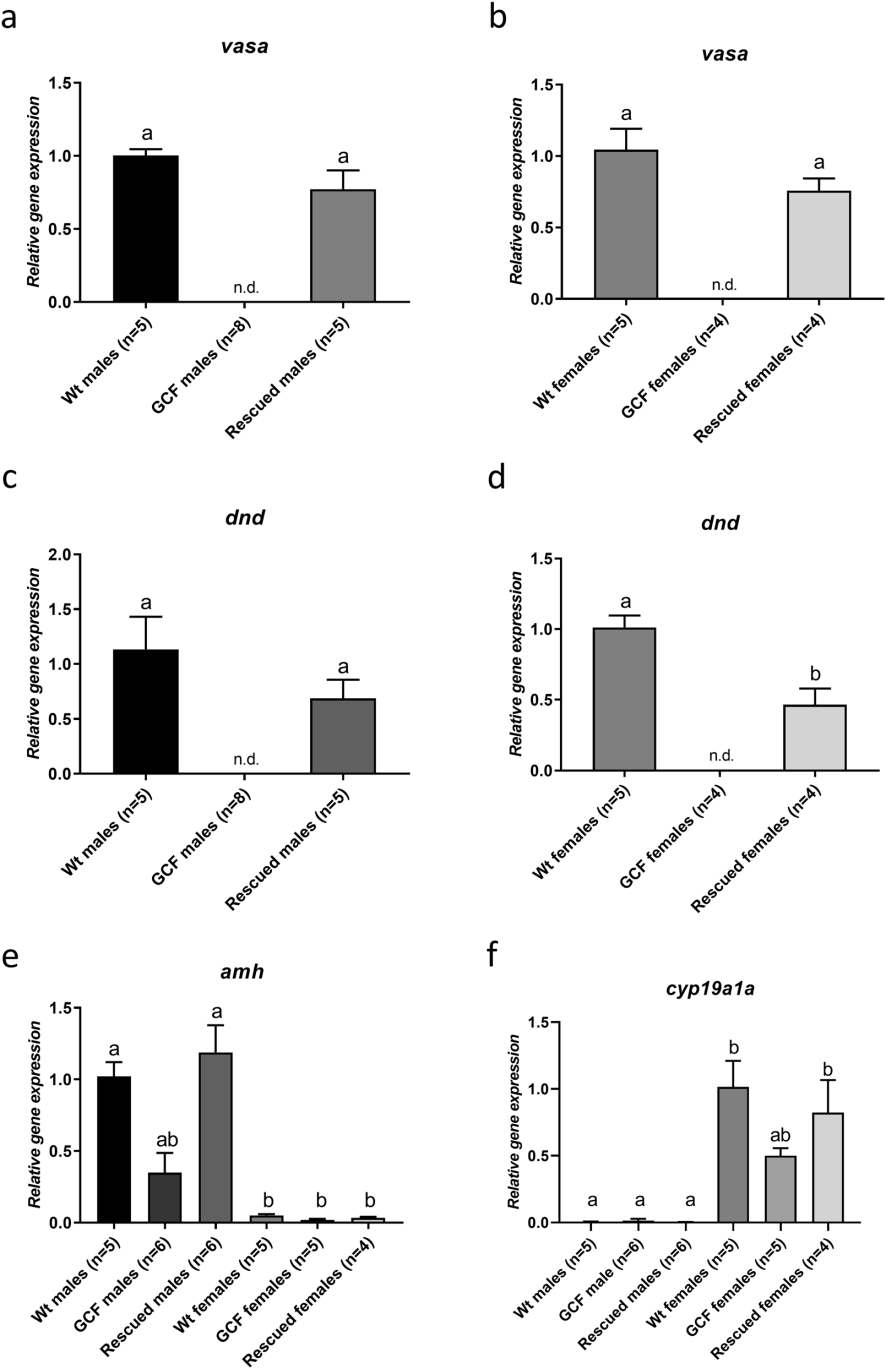
Gene expression in the gonads of *dnd* crispant GCF fish and *dnd* crispant fish with rescued germ cells compared to wt controls. Expression of *vasa* (**a**, **b**), *dnd* (**c**, **d**), *amh* (**e**), *cyp191a* (**f**) relative to *ef1a* in Atlantic salmon testes (**a**, **c**) and ovaries (**b**, **d**) measured by qPCR. Data are shown as mean with SEM, n = 6–8 (GCF males), 5–6 (Rescued males) and 5 (wt males), n = 4–5 (GCF females), 4 (Rescued females) and 5 (wt females). Significant differences between groups are indicated by different letters (a–f). GCF: germ cell-free; Rescued: *dnd* crispant with rescued germ cells; wt: wild type.

To examine the existence of apparently rescued germ cells with other methods, we measured gonadal *vasa* and *dnd* expression in both sexes (Fig. 3a–d). The expression of *vasa* was not significantly different in the immature ovaries and testes of wt and rescued *dnd* crispants; as expected, *vasa* was not detected in GCF gonads (Fig. 3a, b). In rescued *dnd* crispants, *dnd* transcript levels were significantly lower in females but not in males, in comparison to wt gonads; *dnd* expression was not detected in GCF *dnd* crispants (Fig. 3c, d). To confirm the sex of the somatic gonad, *amh* and *cyp19a1a* expression was quantified in male and female somatic gonad respectively (Fig. 3e, f). As expected, expression of *amh* in wt and rescued males was significantly higher than in all females (Fig. 3e), while *cyp19a1a* expression in wt and rescued females was significantly higher than in all males (Fig. 3f).

### Analysis of mutation variants

**Figure 4:**
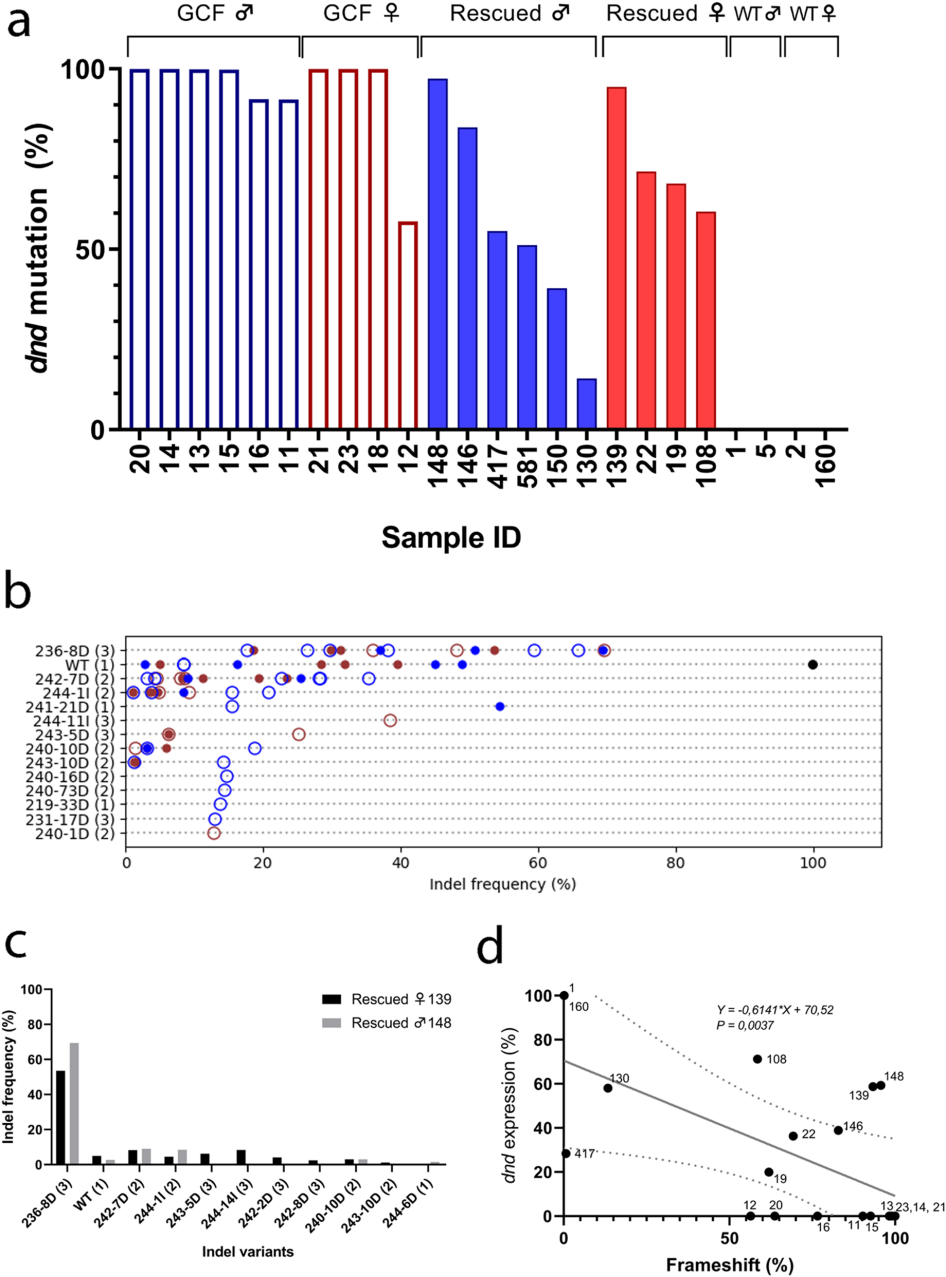
Mutational analysis in the gonad samples of GCF and rescued *dnd* crispant fish compared to wt control fish. (**a**) The mutation rates of *dnd* crispant GCF individuals are shown as framed empty columns in blue for males (11, 13, 14, 20) and in red for females (12, 18, 21, 23); the mutation rates of *dnd* crispant mutants with rescued germ cells are shown as filled columns in blue for males (146, 148, 417, 581) and in red for females (19, 22, 108, 139); the mutation rates of wild type males (1, 5) and wild type females (2, 160) are shown as filled columns in black but not detected, respectively. GCF ♂: testis of *dnd* crispant GCF male; Rescued ♂: testis of *dnd* crispant male with rescued germ cells; GCF ♀: ovary of *dnd* crispant GCF female. rescued ♀: ovary of *dnd* crispant female with germ cell. (**b**) The frequency of each variant (> 10% in at least one individual) from all individuals. The Y-axis lists the variants, showing the position in the reference sequence, the size of the variant, and the type of variant, where D = deletions and I = insertions. The reading frame is indicated by numbers 1–3 in parenthesis. The markers indicate the following: Black = wt, Red = female, Blue = male, open circle = rescued, filled circle = GCF. (**c**) The variants and distribution in fully mutated rescued female 139 and rescued male 148. (**d**) The linear regression analysis shows linear correlation between frameshift mutation and *dnd* expression in rescued males: 148, 146, 130 and 417, rescued females: 139, 19, 22, 108, GCF male: 11, 13, 14, 15, 16, and 20, GCF female: 12, 21 and 23, wt control male: 1, wt control female:160.

CRISPR-Cas9 induced mutations can cause a high degree of mosaicism in several fish species including salmon^26^. To identify the mutated variants in the *dnd* crispants, we deep-sequenced the part of the *dnd* gene containing the CRISPR-induced mutations. The *dnd* amplicons were obtained from gonad tissue sampled from ten rescued *dnd* crispants containing germ cells (4 ♀ and 6 ♂), ten GCF *dnd* crispants (4 ♀ and 6 ♂) and four controls (2 ♀ and 2 ♂) obtained from both experiments (Fig. 4a, Supplementary Table S1 and S2). Individuals from both rescued, and GCF *dnd* crispants, were highly mutated. This included a rescued female (sample ID 139, Figs. 1, 4a) and a rescued male (Figs. 2, 4a, sample ID 148), which had mutation frequencies of 95% and 97% in the gonads (Supplementary Tables S1, S2, and S3), respectively, while at the same time having germ cells (Figs. 1, 2). Seven out of ten deep-sequenced GCF animals displayed a 100% mutation rate (Fig. 4a). All variants and the frequencies of insertions/deletions (indels) among all GCF and rescued samples are shown in Fig. 4b. Depending on their sizes, the different indels affect the reading frame differently, where reading frame (1) does not affect the overall amino acid sequence, while reading frames (2) and (3) represent frameshifts with distorted amino acid sequence downstream of the CRISPR site. Frameshift mutations in reading frames (2) and (3) caused premature termination codons (PTCs) in different locations in the mRNA. The seven most common variants were detected both in GCF and rescued gonads regardless of sex (Fig. 4b). Some variants with mutation frequencies between 10 and 40% were only detected in five GCF gonads (sample ID 18, 15, 16, 20, 21; Fig. 4b, Supplementary Table S4). These specimen-specific frameshift mutations generated PTCs downstream of the CRISPR site in exon 3 or exon 4, depending on the reading frame and the length of the indels (Supplementary Table S4). The most common variant among all mutants was an 8 bp deletion [236-8D (3), TTCCGCTG] causing a shift in the reading frame generating a PTC at aa 159 in exon 4. This variant is shown together with other common variants in fully mutated rescued female 139 and male 148 in Fig. 4c. The second most common variant found in both rescued and GCF fish was a 7 bp deletion [242-7D (2), TGTTCCG] generating an early PTCs at aa 86–87 in the exon 3. 70% of the fish previously characterized as rescued have some degree of wt *dnd* in their genomes (sample ID 19, 22, 108, 146, 417, 581) but so did also 33% of the GCF fish (sample ID 11, 12, 16). As different *dnd* mutation rates in both rescued and GCF *dnd* crispants were observed, we assessed whether there was a correlation between gene expression and frameshift mutation rate in those animals. There was a linear correlation between the rate of frameshift mutation and the expression of *dnd* (deviation from zero is significant, P = 0.0037) that has been demonstrated for rescued and GCF specimens in Fig. 4d. The expression of *dnd* showed linear decrease with a goodness of fit to increase of frameshift rates from the lowest degree mutants to the fully mutated males and females.

**Figure 5:**
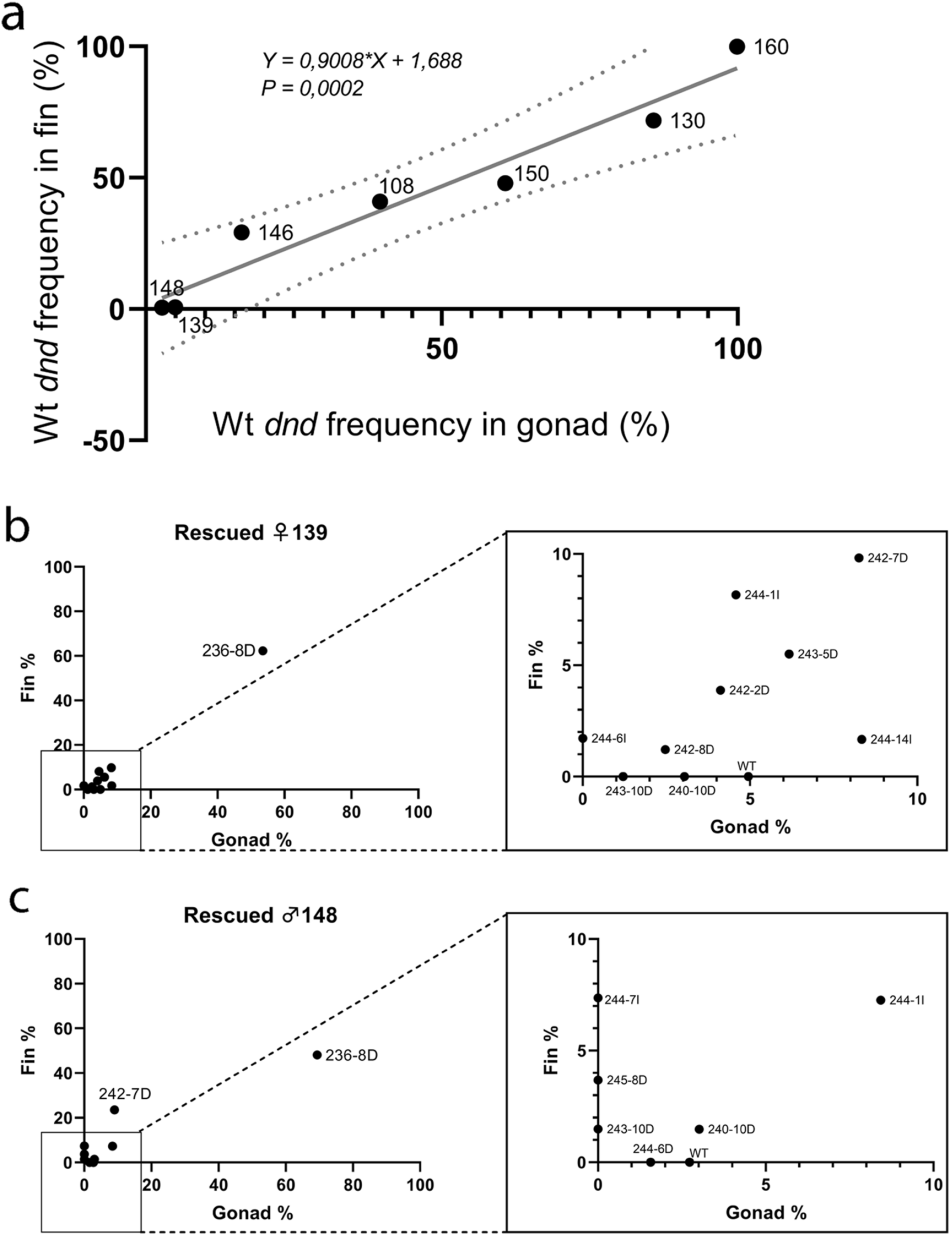
Comparative mutation and variant rates in gonad and fin tissue samples of rescued Atlantic salmon. (**a**) The wt *dnd* frequency correlation between fin and gonad tissues in rescued males: 148, 146, 150, 130, rescued females: 139, 108, and wt control female: 160. (**b**) The rates of each variant in fin and gonad of *dnd* crispant rescued female 139. The variant 243-10D (1.21%) and 240-10D (3.04%) were only detected in the gonad while the variant 244-6I (1.72%) was only found in the fin of rescued female 139. The 4.95 wt% variant was only in the gonad (**c**) The rates of each variant in fin and gonad of *dnd* crispant rescued male 148. The variants in rescued male 148 were 236-8D, 242-7D, 244-1I, 240-10D, 244-6D, 243-10D, 244-7I, 245-8D, wt. We found three variants [243-10D (1.49%), 244-7I (7.36%), 245-8D (3.68%)] specific to fin and the variant 244-6D (1.57%) specific to gonad of rescued male 148, which also carries 2.73 wt%.

The sequenced DNA obtained from fin tissues of all animals are presented in Supplementary Table S1, S2, and S5). The mutation levels in the gonad and fin samples were correlated (deviation from zero is significant, *P* = 0.0002) (Fig. 5a, Supplementary Table S5). The rescued female 139 had three variants including wt which were only detected in the gonad and one variant was only found in the fin (Fig. 5b). The rescued male 148 had three variants specific to fin and two variants including wt were specific to gonad (Fig. 5c). Results indicate that the rescued *dnd* crispants 148 (♂) and 139 (♀) have no wt in fin.

## Discussion

In this study, we have evaluated the initial steps of a method to potentially produce large quantities of sterile, GCF Atlantic salmon. The first step was an apparently paradoxical one, namely, to produce broodstock with germ cells in *dnd* crispants, i.e. produce salmon genetically modified to be GCF that nevertheless are fertile and produce germ cells (Wargelius et al. 2016).

In model fish like zebrafish and medaka, morpholino-based knockdown of the *dnd* transcript results in GCF individuals, while co-injection of *dnd* mRNA and morpholino targeting the *dnd* transcripts can rescue the PGCs^21,27,28^. These studies, however, were not suited to investigate long-term effects beyond the embryonic stages, since morpholino-based knockdown has only transient effects. We have shown here that gonads obtained from one-year old salmon, which were co-injected at the 1-cell stage with a stabilized full-length wt *dnd* mRNA and *dnd* gRNA, had a 97% mutagenesis efficiency in the *dnd* gene and contained rescued germ cells.

The *dnd* gene is highly expressed in adult germ cells in fish including salmon and zebrafish^13,19,29,30^, where specific cellular localization in spermatogonia (sometimes also in spermatocytes) and in previtellogenic oocytes (in medaka also in oogonia) has been observed in several fish species such as medaka, sturgeon, turbot and grouper^31–34^. However, except for the cellular localization, there is no information on Dnd function in adult gonads. Adult zebrafish which express antisense *dnd* in their adult germ cells, display low fertility or sterility, indicating that Dnd protein may have functions in adult germ cells in fish, however possibly this phenotype is also a result of having fewer germ cells due to the transgenic antisense *dnd* expression in primordial and juvenile germ cells^35^. In mice, the adult function of *dnd* has been investigated, with the help of a natural occurring allele of *dnd*, the *Ter* allele. Mice, homozygous for this allele, survive and show lower germ cell counts, infertility and a higher chance of developing testicular cancer^22–24^. In this case, *dnd* is expressed in adult germ cells, but a stop codon has been formed after the RNA recognition motif, resulting in a Dnd protein lacking the ATPase domain. A complete loss of Dnd function in mice results in embryonic lethality, suggesting multiple functions of the protein beyond the germ cells^25^. Interestingly, and in contrast to mice, GCF *dnd* crispant Atlantic salmon can survive and grow^3,19^. Moreover, our study reports that rescued *dnd* crispant Atlantic salmon develop gonads containing germ-cells, indicating the post-embryonic functions of *dnd* are not essential for survival of germ cells in juveniles.

Rescued female *dnd* crispants could not be distinguished from wt controls, based on qualitative histological examination of ovarian sections or the level of *vasa* expression. Rescued *dnd* crispant males, on the other hand, seemed to differ from wt males in terms of the ratio of Sertoli cells over type A spermatogonia, which appeared higher in rescued males. Additional studies, however, are required to confirm this observation by quantitative morphological analyses. Quantifying the transcript level of the germ cell marker *vasa*, did not reveal differences between wt and rescued *dnd* crispants^22,35^. Future work will have to show if the shift in the ratio between Sertoli cells and spermatogonia in favor of Sertoli cells reflects an over-proliferation of the somatic cells, or a reduced or delayed proliferation of germ cells in rescued crispants.

In this study, the CRISPR targeting site in the *dnd* gene is located in exon 3 at aa72 where we detected the expected, variable frameshift mutations in both rescued and GCF *dnd* crispants, with some common variants as previously seen for other CRISPR targeted genes in salmon^26^. The most common variants were found in both GCF and rescued gonads, while some less frequent variants were unique to a tissue or an individual. More importantly, the rate of out-of-frame mutations in the gonads (up to 96%) created a large pool of mutated *dnd* transcripts within the germ cells of these rescued fish. These out-of-frame mutations, most likely activated nonsense-mediated *dnd* mRNA decay as a result of PTCs forming in the mutated *dnd* transcript, detected here as reduced expression of *dnd* in those animals. This observation is also in accordance with what has been observed in other CRISPR studies in salmon^36–39^. The CRISPR-Cas9 cut site in the *dnd* gene, corresponds to aa25 in the 78 aa RNA recognition motif (RRM) in the Dnd protein. The other functional domain in the Dnd protein, the ATPase domain, resides downstream of the cut site. Therefore, it is likely that both domains in a potentially expressed truncated Dnd protein are non-functional.

The efficiency of *dnd* KO was up to 99.9% in GCF and 97.3% in rescued salmon gonads, respectively while they all displayed 99.9% mutation rates in their fins. Hence, *dnd* mutation frequencies in gonad and fin samples were significantly correlated. However, two of the GCF individuals contained a high percentage of wt *dnd* reads (sample ID 12, 45% and sample ID 11, 12%) while still being GCF. We assume that this setting reflects a mosaic pattern of mutations in F0 fish. In these mutants, all the PGCs were probably fully mutated for the *dnd* gene and therefore could not survive as PGC^19,40^, while somatic cells still may hold wt *dnd* in their genomes without affecting survival of germ cells. This also may have been the case in the rescued *dnd* crispant salmon gonads displaying between 3 and 50 wt% *dnd*, although in these fertile mutants, there may have been some wt *dnd* expressed in germ cells. It is, however, unlikely that such a large proportion of *dnd* mutations would only be present in somatic cells, since germ cells are abundant in fish gonads, especially in male testis of this stage where 20–25% of all cells are germ cells^41–43^. If we had germ cells with wt *dnd* expression scattered in the gonads of rescued fish, we should probably also have seen what we have observed previously in some male *dnd* crispants, displaying loss of germ cells in one area of the testes, while other areas within the same tissue contained germ cells^19^. This phenotype was not observed in any of our rescued male mutants. To confirm homozygous mutations in the whole gonad, it is possible to cross rescued *dnd* crispants and obtain homozygous mutants for the most common *dnd* loss of function variant, i.e. the variant 236-8D. Although the most common variants were found in both GCF and rescued fish gonads, the mosaicism in rescued fish was higher than in GCF fish. Some variants were unique to tissue (fin or gonad) and an individual, however, these specimen-specific variants were not depending on being GCF or sex. The fact that the mutation rate is very high (97.3%) in the *dnd*-KO rescued fish gonads in both females and males holds great promise to be a successful application of the method. Overall, our results indicate that addition of wt *dnd* mRNA prevents PGCs from transdifferentiating to somatic cells^19,40^, allowing the establishment of a germ cell population in both testicular and ovarian tissue in *dnd* crispant salmon. The high efficiency of *dnd* knockout in rescued germ cells suggests that offspring of these founders will be sterile. However, the ability of the rescued germ cells carrying mutated *dnd* sequences to develop into functional sperm or eggs in salmon remains to be demonstrated.

The method presented here is complementary to other already developed transgenic and knockdown methods to produce sterile fish for aquaculture^11^. However, in contrast to other non-transgenic methods like embryo immersion in *dnd* Vivo-conjugated morpholino^44^ or triploidization^10^, our approach if functional in all steps display a significant advantage since it will always produce 100% sterility in combination with the transmissibility of sterility to the next generation. One of the significant advantages of this method is that it can be applied to both sexes without additional steps such as germ cell transplantation or sex reversal^45^. The long reproductive cycle makes the implementation and standardization of the procedure time-dependent; however, after creating the broodstock, microinjections of *dnd* mRNA in *dnd* crispant offspring will enable further restocking. As a conclusion, we report the first step of a procedure to generate sterility in production stocks of salmon while preserving the fertility of the breeding nucleus. This feature would help protect the genetic integrity of wild salmon populations, prevent negative welfare effects otherwise associated with sexual maturation, thereby further increasing the sustainability of salmon farming. Moreover, implementing this approach would also simplify protecting IPRs.

Currently, we are examining the maturation in rescued males and females, which will take another year in females, so that generation of F1 homozygous and heterozygous mutation carriers can take place in 2021. The generation of sterile offspring will take four years in our facility but allows obtaining thousands of sterile larvae, which can be used for different purposes such as surrogate production of endangered or valuable fishes or aquaculture without threatening the wild population of salmon. In this regard, further studies will focus on examining the quality of rescued gametes, as well as investigating the germline transmission of *dnd* mutations, and will be followed by examinations of phenotypes and genotypes of juveniles in the F1 generation.

## Materials and methods

### Production of CRISPR-Cas9 constructs and full-length *dnd* mRNA

CRISPR oligos targeting *slc45a2* and *dead end* (*dnd*) were annealed, cloned, linearized as previously described^19,26^. Different from previous studies, *in-vitro* transcription was conducted with the HiScribe T7 High Yield RNA Synthesis Kit (NEB) according to the manufacturer’s protocol and template DNA was eliminated by incubation with 1 µl TURBO DNase (Ambion) at 37 °C for 15 min. The gRNAs were purified using RNeasy column (Qiagen) according to the manufacturer’s instructions. Target sites of both *slc45a2* and *dnd* were selected to minimize potential off-target effects as described in Wargelius et al.^19^. *Cas9* mRNA was prepared by in vitro transcription as described previously for zebrafish^46^. We used QIAprep Spin Miniprep Kit (Qiagen) for the isolation, the mMessage mMachine T3 kit (Ambion) for *in-vitro* transcription, and RNeasy column (Qiagen) for purification according to the manufacturers’ instructions.

Full-length *dnd* mRNA was generated by PCR amplification of ovarian cDNA from Atlantic salmon using Q5 High-Fidelity DNA polymerase (NEB) and a forward primer that included a T7 promoter 5′-GAT TTA ATA CGA CTC ACT ATA GGG AAC AGA CCA CCA TGG AGG AGC GTT CAA GTC AGC AGG -3′ with a reverse primer 5′-TTT GAC AAA TCT CAT TTT ATT ATA ATG AGA AAC AA-3′. The PCR product was extracted from a 1% agarose gel, purified with QIAquick Gel Extraction Kit (Qiagen), and sequenced by Sanger sequencing. The *dnd* PCR product was transcribed in vitro into a functional 5′-capped and polyadenylated *dnd* mRNA using the HiScribe T7 ARCA mRNA Kit (NEB).

### Preparation of embryos

Atlantic salmon eggs and sperm were obtained from AquaGen AS (Trondheim, Norway). The eggs were fertilized and treated with 0.5 mM L-glutathione reduced (Sigma-Aldrich) solution (pH 10) to prevent chorion hardening^47^. Fertilized eggs were then incubated for 3–4 h at 6–8 °C to allow for blastodisc formation, at which stage the eggs were used for microinjection.

### Experimental design and microinjections

The microinjection experiments took place at the Institute of Marine Research facilities at Matre Aquaculture Research station, Norway. The first rescue experiment was carried out in 2016. In this experiment, we injected two gRNAs targeting the *dnd* and *slc45a2* genes (resulting in an albino phenotype of *dnd* crispant animals^26^, facilitating visual screening) together with the full length 5′-capped and polyadenylated wild type (wt) *dnd* mRNA to rescue the germ cells into the fertilized salmon embryos (n = 259) at the 1-cell stage of embryo development^19,26^. Embryos were injected with a mixture of 50 ng/ml *dnd* gRNA, 50 ng/ml *slc45a2* gRNA, 150 ng/ml *Cas9* mRNA and 100 ng/ml *dnd* full length mRNA in nuclease-free water, using the FemtoJet 4i (Eppendorf) microinjector with a pressure of 100 hPa for 1 s and micro-needles from Narishige (Japan). After injection, embryos were transferred to hatchery trays and incubated in running fresh water (2.5 L/min.kg fish) at 8 °C until hatching, subsequently were transfer to the indoor tanks (size:1 × 1 m, density:100 kg/m^3^). Fish were fed a standard commercial feed. We sampled gonads of 13 fully albino rescued fish, six females and seven males from the surviving one-year old rescued fish (n = 79), and 2 wt controls, i.e. one male and one female. One whole gonad of each fish was stored in RNAlater (Ambion) for RNA extraction to use for gene expression analysis while the other gonad was fixed in Bouin’s solution for histological analysis. At 15 months old, the remaining fish were anesthetized with benzocaine (0,1 g/L) and then intraperitoneally tagged with 8 mm passive inductive transponder-tags (ID-100 A Microtransponder, Trovan Ltd) after anesthetization using benzocaine (0,1 g/L). Weight, length and the skin coloration phenotype of all tagged fish were recorded. In addition, adipose fin tissue samples were collected in 99.9% ethanol for DNA extraction to be used for mutation analysis and genotypic sexing. At 16 months old, we sampled five fully albino rescued fish, one female and four males along with two wt controls, one male and one female.

The second rescue experiment was carried out in November 2017. We injected one gRNA targeting *dnd* and *Cas9* mRNA together with *dnd* mRNA into fertilized group of 1108 salmon embryos, as described above. They were kept under hatchery conditions as described earlier together with an untreated control group. At nine mpf, fin samples were collected from 60 fish and of five wt control fish to screen for mutations using HRM analysis followed by sequencing and qPCR analysis. At 11 mpf, gonad samples of the identified mutants were collected, and the remaining injected and untreated control fish were tagged, measured (weight and length), and fin clips were obtained for mutation analysis and subsequent sexing. The fish have been kept for the future studies up until expected spawning in autumn 2020.

### Screening for mutations

#### High resolution melt (HRM) analysis

Genomic DNA was prepared from fin clips of the experimental groups (Rescue-1, n = 30; wt control, n = 5) from 2017 by use of the Hotshot method^48^. Briefly samples were heated in 100 µl 50 mM NaOH at 95 °C for 20 min. The reaction was then neutralized by adding 10 µl 1 M Tris–HCl pH 7,5. 2 µl of a 1:4 dilution of DNA template was used for a 10 µl HRM reaction volume using PowerUp SYBR Green Master Mix (Applied Biosystems) according to the protocol of the manufacturer. The thermocycler reaction proceeded on an HRM capable real-time PCR machine (Applied Biosystems), where the final melt curve step was programmed with a ramp rate of 0.015 °C/s for 95 °C. A pair of oligonucleotides were used for *dnd* (GenBank accession: NC_027304.1) PCR; forward 5′-TACATGCATCATTCCCACCCCA-3′ and reverse 5′-AAGTTCCACCATTACACTGCTT-3′ giving a 542 bp PCR product as described previously^19^. These gene specific primers span the intron–exon junction in exon3, where the CRISPR target site is placed. Fish carrying CRISPR indels were automatically separated from non-mutants by the shape of the normalized melt curves utilizing an unsupervised hdbscan clustering approach^49^.

#### Sanger Sequencing of mutants

Genomic DNA was extracted from the fin clips with the DNeasy 96 Blood and Tissue Kit (Qiagen) according to the manufacturer’s protocol. PCR was carried out using Colorless GoTaq Flexi Reaction Buffer (Promega) with PCR *dnd* primers^19^. The PCR product was purified with QIAquick PCR purification Kit (Qiagen) and measured via Qubit Fluorometric Quantitation (Invitrogen). Purified and quantified PCR products were cloned into PCR4-TOPO using the TOPO TA cloning kit for sequencing (Invitrogen). Cloned fragments were sequenced with M13 forward and reversed primers using BigDye 3.1.

#### Deep sequencing

Genomic DNA from fin clips purified using Qiagen DNA extraction protocol were used as template for a two-step barcoding PCR targeting the CRISPR locus as described by Gagnon et al.^50^. Briefly, each sample was given a unique 6-mer barcode (Trueseq barcode primers) where after the barcoded *dnd* PCR products were mixed in equimolar ratios (oligos forward: 5′ tct ttc cct aca cga cgc tct tcc gat ctC TAC ATG CAT CAT TC CCA CCC CA 3′, reverse: 5′ tgg agt tca gac gtg tgc tct tcc gat ctA AG TTC CAC CAT TAC ACT GCT T 3′). We used the same PCR primers as described in the above HRM protocol. The final denatured sequencing library was prepared at a concentration of 8 pM and spiked with 5% denatured phiX and sequenced on the MiSeq using MiSeq Reagent Kit v3 (600 cycle format).

### Mutation and variant analysis

The reads obtained from deep sequencing, were filtered to only retain reads starting with the primer sequences (allowed 1 nt mismatch in each primer sequence). The sequenced *dnd* amplicons obtained from forward and reverse template sequencing overlapped with 58 bp in their 3′ ends, which allowed the reads to be merged if the overlap was at least 40 bp with at most 8 mismatches. For each overlapping base, the nucleotide having the highest base quality was used, yielding single forward reads covering the entire amplicon. These reads were cropped to only include positions 200–300 in each read, covering the *dnd* CRISPR site. Identical sequences were grouped together, and unique sequences represented by only one single read, were discarded to reduce noise from sequencing errors. The filtered sequences were aligned one-by-one to the amplicon reference sequence using Muscle (PMID: 15034147). Indels were identified and quantified for each sample and were given names reflecting the 5′ position of the indel in the amplicon sequence, and the size and type of indel (D = deletion, I = insertion). Reading frames for each indel combination was determined by the function 1 + [size (D)-size (I)] modulo 3, where size (D) and size (I) are the sizes of the identified deletions and insertions, respectively, in a given sequence. The information about reading frames was used to calculate the proportions of in-frame and frameshift mutations in each sample. Indel variants represented by at least 1% of the total reads in a sample.

### Relative gene expression analysis

Total RNA was isolated from the gonad samples using a RNeasy Mini Kit (Qiagen), and DNase treated by TURBO DNase (Ambion) according to the manufacturers’ instructions. The RNA concentration was determined by NanoDrop NP-1000 spectrophotometer (NanoDrop technologies, Wilmington, DE, USA). cDNA was synthesized with SuperScript VILO cDNA Synthesis Kit (Invitrogen) by following the protocol of the manufacturer. qRT-PCR reactions were set up for four replicates and run with *ef1a* (as a reference gene), for *dnd* using the reagents of PowerUp SYBR Green Master Mix (Applied Biosystems); for *vasa*, *cyp19a1a* and *amh* TaqMan Universal PCR Master Mix (Applied Biosystems) according to the protocol of the manufacturer. Primers and probes are listed in Supplementary file of our previous work^19^. The relative gene expressions were calculated by applying the method of Comparative Ct (or 2^−ΔΔCt^) (Applied Biosystems). The data was calibrated to the sample with the highest gene expression.

### Genetic sex identification

To identify the genetic sex of all experimental fish, we used the double exon of *sdY* (exon2 and exon4) assay, to be able to select genetic females and males to compare with histologic analysis^51,52^.

### Histological analysis

Gonad samples were fixed in Bouin’s solution overnight at 8 °C and stored in 80% ethanol until tissue processing. Subsequently, the samples were dehydrated, and embedded in paraffin blocks that served, to produce 5-µm-thick sections, according to conventional techniques. After deparaffinization and rehydration, the sections were stained with hematoxylin–eosin. The sections were documented using a digital slide scanner C13210-10 NanoZoomer S60 (Hamamatsu Photonics K.K.) equipped with a 40× lens and a 10× ocular, i.e. at 400-fold magnification. Digitized slides were analyzed qualitatively for the developmental stage of the germ cells and the presence of somatic cell types.

### Statistical analysis

Statistics were performed using GraphPad Prism8. Due to the low number of replicate qPCR samples, none of the samples showed a normal distribution (D’Agostino and Pearson omnibus normality test). Hence, a nonparametric Kruskal–Wallis followed by a two-stage linear step-up procedure of Benjamini Krieger and Yekutieli post-test was applied for *cyp19* and *amh* expression data groups. An unpaired two-tailed t-test (Kolmogorov smirnov test) was performed to determine if significant differences existed for *vasa* and *dnd* expression between wt and rescued *dnd* crispant gonads. Data are presented as average ± standard error of the mean. Simple linear regression analyses were also employed to test for a trend, such as a decrease or a correlation.

### Ethics

The use of experimental animals complied with the Norwegian Animal Welfare Act of 19th of June 2009, in force from 1st of January 2010. All experiments performed in this study were approved by the Norwegian Animal Research Authority (https://www.fdu.no/fdu/), NARA, permit number 5741.

## Supplementary information

Supplementary Tables.

Supplementary Figure Legends.

Supplementary Figure 1.

Supplementary Figure 2.

Supplementary Figure 3.

Supplementary Figure 4.

Supplementary Figure 5.
