# Author Correction: Rescue of germ cells in *dnd* crispant embryos opens the possibility to produce inherited sterility in Atlantic salmon

**DOI:** 10.1038/s41598-021-86045-0

**Published:** 2021-03-22

**Authors:** Hilal Güralp, Kai O. Skaftnesmo, Erik Kjærner‑Semb, Anne Hege Straume, Lene Kleppe, Rüdiger W. Schulz, Rolf B. Edvardsen, Anna Wargelius

**Affiliations:** 1 Institute of Marine Research, Bergen, Norway; 2 Faculty of Fisheries and Protection of Waters, South Bohemian Research Center of Aquaculture and Biodiversity of Hydrocenoses, University of South Bohemia in Ceske Budejovice, Zátiší 728/II, 389 25 Vodňany, Czech Republic; 3 Department of Biology, Faculty of Science, Utrecht University, Padualaan 8, 3584 CH Utrecht, The Netherlands

Correction to: *Scientific Reports* 10.1038/s41598-020-74876-2, published online 22 October 2020

This Article contains a typographical error in the Materials and methods section under subheading ‘Experimental design and microinjections,’ where

“Embryos were injected with a mixture of 50 ng/ml *dnd* gRNA, 50 ng/ml *slc45a2* gRNA, 150 ng/ml *Cas9* mRNA and 100 ng/ml *dnd* full length mRNA in nuclease-free water, using the FemtoJet 4i (Eppendorf) microinjector with a pressure of 100 hPa for 1 s and micro-needles from Narishige (Japan).”

should read:

“Embryos were injected with a mixture of 50 ng/μl *dnd* gRNA, 50 ng/μl *slc45a2* gRNA, 150 ng/μl *Cas9* mRNA and 100 ng/μl *dnd* full length mRNA in nuclease-free water, using the FemtoJet 4i (Eppendorf) microinjector with a pressure of 100 hPa for 1 s and micro-needles from Narishige (Japan).”

