# Supplementary material for: Rescue of germ cells in dnd crispant embryos opens the possibility to produce inherited sterility in Atlantic salmon
Source: Sci Rep. 2020 Oct 22;10:18042. doi: 10.1038/s41598-020-74876-2 (PMC7581530; doi:10.1038/s41598-020-74876-2)
Supplement: Supplementary file 1 — Supplementary Tables. [file 41598_2020_74876_MOESM1_ESM.docx]

Supplementary Tables:

**Rescue of germ cells in *dnd* crispant embryos opens the possibility to produce inherited sterility in Atlantic salmon**

Authors: Hilal Güralp^1^, Kai O. Skaftnesmo^1^, Erik Kjærner-Semb^1^, Anne Hege Straume^1^, Lene Kleppe^1^, Rüdiger W. Schulz^1,2^, Rolf B. Edvardsen^1^, Anna Wargelius^1*^

Affiliations: ^1^ Institute of Marine Research, Bergen, Norway

^2^ Utrecht University, Faculty of Science, Department of Biology, Padualaan 8, 3584 CH, Utrecht, The Netherlands.

*Corresponding author e-mail: [anna.wargelius@imr.no](mailto:anna.wargelius@imr.no)

Supplementary Table S1. Detailed information on samples from the experiment in 2016.

| ID | Biogroup | sex | External Phenotype | Gonad Histolgy | Sampling Date | Body Weight (g) | Length (cm) | Wt *dnd* Frequencies % |
| --- | --- | --- | --- | --- | --- | --- | --- | --- |
| 1 | wt | M | Normal | N | 9.11.2017 | 44.5 | 16.5 | 99.87 |
| 2 | wt | F | Normal | N | 9.11.2017 | 38.6 | 15 | 99.85 |
| 3 | wt | M | Normal | N | 9.3.2018 | 17.5 | 58 | NA |
| 5 | wt | F | Normal | N | 9.3.2018 | 150 | 23 | NA |
| 11 | *dnd/alb* crispant | M | Albino | GCF | 9.11.2017 | 33.2 | 14 | 8.42 |
| 12 | *dnd/alb* crispant | F | Albino | GCF | 9.11.2017 | 50.4 | 16.5 | 42.29 |
| 13 | *dnd/alb* crispant | M | Albino | GCF | 9.11.2017 | 29.5 | 13.6 | 0.16 |
| 14 | *dnd/alb* crispant | M | Albino | GCF | 9.11.2017 | 14.2 | 11 | 0.03 |
| 15 | *dnd/alb* crispant | M | Albino | GCF | 9.11.2017 | 24.1 | 12.5 | 0.24 |
| 16 | *dnd/alb* crispant | M | Albino | GCF | 9.11.2017 | 11.3 | 10 | 8.36 |
| 17 | *dnd/alb* crispant | M | Mosaic | tissue lost | 9.11.2017 | 16.1 | 11.2 | NA |
| 18 | *dnd/alb* crispant | F | Albino | GCF | 9.11.2017 | 42.4 | 14.4 | 0.02 |
| 19 | Rescued *dnd/alb* crispant | F | Albino | N | 9.11.2017 | 55.2 | 16.8 | 31.80 |
| 20 | *dnd*/alb crispant | M | Albino | GCF | 9.11.2017 | 72.9 | 18.5 | 0.01 |
| 21 | *dnd/alb* crispant | F | Albino | GCF | 9.11.2017 | 45.1 | 15.9 | 0.01 |
| 22 | Rescued *dnd/alb* crispant | F | Albino | N | 9.11.2017 | 48.1 | 16.1 | 28.45 |
| 23 | *dnd/alb* crispant | F | Mosaic | GCF | 9.11.2017 | 36.4 | 14.7 | 0.02 |
| 28 | *dnd/alb* crispant | M | Albino | GCF | 9.3.2018 | 27 | 13 | 0.08 * |
| 34 | *dnd/alb* crispant | F | Albino | GCF | 9.3.2018 | 30 | 14 | 0.08* |
| 39 | *dnd/alb* crispant | M | Albino | GCF | 9.3.2018 | 56 | 16.5 | 0.05* |
| 58 | Rescued *dnd/alb* crispant | M | Albino | N | 9.3.2018 | 83 | 19 | 42.70* |
| 83 | Rescued *dnd/alb* crispant | M | Albino | N | 9.3.2018 | 67 | 16 | 0.02* |

(wt: wild type, N: normal, GCF: germ cell free, *: in fin, NA: not analyzed)

Supplementary Table S2. Detailed information on samples from the experiment in 2017.

| Sample ID | Biogroup | Sex | Gonad (GCF/N) | Body Weight (g) | Length (cm) | Wt *dnd* Frequencies % in Gonad | Wt *dnd* Frequencies % in Fin |
| --- | --- | --- | --- | --- | --- | --- | --- |
| 108 | Rescued *dnd* crispant | F | N | 112 | 20 | 39.56 | 40.92 |
| 110 | Rescued *dnd* crispant | M | Lost fish | NA | NA | NA | 25.13 |
| 130 | Rescued *dnd* crispant | M | N | 89 | 18.5 | 85.81 | 71.76 |
| 139 | Rescued *dnd* crispant | F | N | 104 | 19 | 4.95 | 0.69 |
| 146 | Rescued *dnd* crispant | M | N | 55 | 16 | 16.20 | 29.21 |
| 148 | Rescued *dnd* crispant | M | N | 136 | 21.5 | 2,73 | 0.58 |
| 150 | Rescued *dnd* crispant | M | N | 139 | 20.5 | 60.77 | 47.92 |
| 417 | Rescued *dnd* crispant | M | N | 112 | 20.3 | 44.94 | 14.52 |
| 581 | Rescued *dnd* crispant | M | N | 135 | 22.4 | 48.90 | 14.46 |
| 160 | Wild type | F | N | 132 | 22.5 | 99.90 | 99.9 |
| 164 | Wild type | M | N | 69 | 17 | NA | 99.88 |
| 161 | Wild type | M | N | 100 | 20,5 | NA | NA |
| 162 | Wild type | M | N | 104 | 21 | NA | NA |
| 163 | Wild type | F | N | 115 | 21,5 | NA | NA |

(N: normal, GCF: germ cell free, NA: not analyzed)

Supplementary Table S3. The proportion of wt, in-frame and frameshift in total reads in gonad.

| **Sample ID** | **Biogroup** | **WT (%)** | **In-frame (%)** | **Frameshift (%)** | **Total reads** |
| --- | --- | --- | --- | --- | --- |
| 1 | Wild type | 99.87 | 0.01 | 0.12 | 267851 |
| 2 | Wild type | 99.85 | 0.02 | 0.13 | 354275 |
| 5 | Wild type | 99.93 | 0.01 | 0.06 | 21413 |
| 160 | Wild type | 99.9 | 0 | 0.1 | 18926 |
| 108 | Rescued | 39.56 | 1.99 | 58.45 | 13138 |
| 130 | Rescued | 85.81 | 0.81 | 13.38 | 16383 |
| 139 | Rescued | 4.95 | 1.76 | 93.29 | 13021 |
| 146 | Rescued | 16.2 | 0.92 | 82.88 | 15721 |
| 148 | Rescued | 2.73 | 1.59 | 95.67 | 18696 |
| 150 | Rescued | 60.77 | 0.36 | 38.87 | 16247 |
| 417 | Rescued | 44.94 | 54.37 | 0.68 | 18338 |
| 581 | Rescued | 48.9 | 0 | 51.1 | 14375 |
| 11 | GCF | 8.42 | 1.33 | 90.25 | 38825 |
| 12 | GCF | 42.29 | 1.32 | 56.39 | 227 |
| 13 | GCF | 0.16 | 1.62 | 98.23 | 40694 |
| 14 | GCF | 0.03 | 0.03 | 99.95 | 35592 |
| 15 | GCF | 0.24 | 7.14 | 92.61 | 37983 |
| 16 | GCF | 8.36 | 15.05 | 76.59 | 33764 |
| 18 | GCF | 0.02 | 2.12 | 97.86 | 36204 |
| 19 | GCF | 31.8 | 6.28 | 61.92 | 379696 |
| 20 | GCF | 0.01 | 36.27 | 63.72 | 29654 |
| 21 | GCF | 0.01 | 1.04 | 98.96 | 28468 |
| 22 | GCF | 28.45 | 2.3 | 69.25 | 290294 |
| 23 | GCF | 0.02 | 0.04 | 99.94 | 323953 |
| 34* | GCF | 0.08 | 0.21 | 99.71 | 25935 |

GCF: germ cell-free *dnd* crispant, Rescued: rescued *dnd* crispant, *: fin

Supplementary Table S4. Unique indel variants in germ cell free gonads and positions of the PTCs. (represented by at least 1% of the total reads per sample)

| **Sample ID** | **Indel** | **Reading frame** | **Reads in gonad** | | **Position of PTC** | |
| --- | --- | --- | --- | --- | --- | --- |
|  |  |  | **quantity** | **rate** | **exon** | **aa** |
| **18** | 244-11I | 3 | 13897 | 38.39% | 3 | 86-87 |
| **15** | 240-16D | 2 | 5582 | 14.7% | 3 | 86-87 |
| **15** | 231-17D | 3 | 4906 | 12.92% | 4 | 159 |
| **16** | 240-73D | 2 | 4818 | 14.27% | 4 | 159 |
| **20** | 219-33D | 1 | 4050 | 13.66% | - | - |
| **21** | 240-1D | 2 | 3646 | 12.81% | 3 | 86-87 |

I: insertion, D: deletion, aa: aminoacid, PTC: premature termination codon

Supplementary Table S5. Comparative indel variants in gonad and fin represented by at least 1% of the total reads per sample.

| **Sample ID** | **Indel** | **Reading frame** | **Reads in gonad** | | **Reads in fin** | |
| --- | --- | --- | --- | --- | --- | --- |
|  |  |  | **quantity** | **rate** | **quantity** | **rate** |
| **108** | WT | 1 | 5197 | 39.56% | 110541 | 40.92% |
| **108** | 236-8D | 3 | 4092 | 31.15% | 84214 | 31.17% |
| **108** | 242-7D | 2 | 3082 | 23.46% | 62428 | 23.11% |
| **108** | 244-10I | 2 | 341 | 2.6% | 0 | 0% |
| **139** | 236-8D | 3 | 6966 | 53.5% | 237844 | 62.31% |
| **139** | WT | 1 | 645 | 4.95% | 0 | 0% |
| **139** | 242-7D | 2 | 1075 | 8.26% | 37502 | 9.82% |
| **139** | 244-1I | 2 | 596 | 4.58% | 31150 | 8.16% |
| **139** | 243-5D | 3 | 804 | 6.17% | 21007 | 5.5% |
| **139** | 244-14I | 3 | 1086 | 8.34% | 6374 | 1.67% |
| **139** | 242-2D | 3 | 537 | 4.12% | 14793 | 3.88% |
| **139** | 242-8D | 3 | 322 | 2.47% | 4625 | 1.21% |
| **139** | 240-10D | 2 | 396 | 3.04% | 0 | 0% |
| **139** | 244-6I | 1 | 0 | 0% | 6563 | 1.72% |
| **139** | 243-10D | 2 | 158 | 1.21% | 0 | 0% |
| **146** | WT | 1 | 2547 | 16.2% | 94419 | 29.21% |
| **146** | 242-7D | 2 | 4010 | 25.51% | 122849 | 38.01% |
| **146** | 236-8D | 3 | 5813 | 36.98% | 59778 | 18.49% |
| **146** | 232-32D | 3 | 1493 | 9.5% | 5388 | 1.67% |
| **146** | 244-2D | 3 | 871 | 5.54% | 0 | 0% |
| **146** | 242-8D | 3 | 225 | 1.43% | 6517 | 2.02% |
| **146** | 244-1I | 2 | 0 | 0% | 6316 | 1.95% |
| **146** | 243-10D | 2 | 0 | 0% | 4324 | 1.34% |
| **146** | 244-14I | 3 | 0 | 0% | 3709 | 1.15% |
| **146** | 228-18D | 1 | 0 | 0% | 3628 | 1.12% |
| **148** | 236-8D | 3 | 12970 | 69.37% | 149441 | 48.12% |
| **148** | WT | 1 | 511 | 2.73% | 0 | 0% |
| **148** | 242-7D | 2 | 1685 | 9.01% | 73001 | 23.51% |
| **148** | 244-1I | 2 | 1577 | 8.43% | 22560 | 7.26% |
| **148** | 244-7I | 2 | 0 | 0% | 22868 | 7.36% |
| **148** | 240-10D | 2 | 565 | 3.02% | 4601 | 1.48% |
| **148** | 245-8D | 3 | 0 | 0% | 11422 | 3.68% |
| **148** | 244-6D | 1 | 294 | 1.57% | 0 | 0% |
| **148** | 243-10D | 2 | 0 | 0% | 4637 | 1.49% |
| **417** | WT | 1 | 8242 | 44.94% | 4905 | 14.52% |
| **417** | 241-21D | 1 | 9955 | 54.29% | 0 | 0% |
| **417** | 236-8D | 3 | 0 | 0% | 14044 | 41.57% |
| **417** | 242-7D | 2 | 0 | 0% | 6036 | 17.87% |
| **417** | 236-197D | 3 | 0 | 0% | 2267 | 6.71% |
| **417** | 244-1I | 2 | 0 | 0% | 1581 | 4.68% |
| **417** | 240-1I4M1I | 3 | 0 | 0% | 712 | 2.11% |
| **417** | 218-43D | 2 | 0 | 0% | 616 | 1.82% |
| **417** | 244-11I | 3 | 0 | 0% | 567 | 1.68% |
| **417** | 240-17D | 3 | 0 | 0% | 540 | 1.6% |
| **417** | 241-36D | 1 | 0 | 0% | 439 | 1.3% |
| **417** | 244-6D | 1 | 0 | 0% | 424 | 1.25% |
| **417** | 243-10D | 2 | 0 | 0% | 372 | 1.1% |

I: insertion, D: deletion, M: match/mismatch, WT: wild type
