# Supplementary material for: Rescue of germ cells in dnd crispant embryos opens the possibility to produce inherited sterility in Atlantic salmon
Source: Sci Rep. 2020 Oct 22;10:18042. doi: 10.1038/s41598-020-74876-2 (PMC7581530; doi:10.1038/s41598-020-74876-2)
Supplement: Supplementary file 2 — Supplementary Figure Legends. [file 41598_2020_74876_MOESM2_ESM.docx]

Supplementary Figure legends:

**Rescue of germ cells in *dnd* crispant embryos opens the possibility to produce inherited sterility in Atlantic salmon**

Authors: Hilal Güralp^1^, Kai O. Skaftnesmo^1^, Erik Kjærner-Semb^1^, Anne Hege Straume^1^, Lene Kleppe^1^, Rüdiger W. Schulz^1,2^, Rolf B. Edvardsen^1^, Anna Wargelius^1*^

Affiliations: ^1^ Institute of Marine Research, Bergen, Norway

^2^ Utrecht University, Faculty of Science, Department of Biology, Padualaan 8, 3584 CH, Utrecht, The Netherlands.

*Corresponding author e-mail: [anna.wargelius@imr.no](mailto:anna.wargelius@imr.no)

**Supplementary Fig. S1.** Gross morphology and histology of 1-year-old females. Sampling date: 9.11.2017; 2: wt control; 12, 18, 21, 23: *dnd* crispant germ cell free; 19 and 22: *dnd* crispant with germ cells; Scale bar: 50µm.

**Supplementary Fig. S2.** Gross morphology and histology of 1-year-old-males. Sampling date: 9.11.2017; 1: wt control; 11, 13, 14, 15, 16 and 20: *dnd* crispant germ cell free;
Scale bar: 50µm.

**Supplementary Fig. S3.** Gross morphology and histology of 16-months-old males and females.
Sampling date: 8 March 2018; 3: wt control male; 28, 39: *dnd* crispant germ cell free males; 58, 83: *dnd* crispant males with germ cells; 5: wt control female; 34: *dnd* crispant germ cell free female; Scale bar: 50µm.

**Supplementary Fig. S4.** Gross morphology and histology of 11-months-old females. Fertilization date:10 November 2017; Sampling date: 11 October 2018; 160: wt control; 108, 139: *dnd* crispant with germ cells; Scale bar: 100 µm

**Supplementary Fig. S5.** Gross morphology and histology of 11-months-old males. Fertilization date:10 November 2017; Sampling date: 11 October 2018; 162: wt control; 130, 146, 148, 150: *dnd* crispant with germ cells; Scale bar: 50 µm
